# Artificial Intelligence: A Universal Virtual Tool to Augment Tutoring in Higher Education

**DOI:** 10.1155/2022/1410448

**Published:** 2022-05-09

**Authors:** K. Hemachandran, Priti Verma, Purvi Pareek, Nidhi Arora, Korupalli V. Rajesh Kumar, Tariq Ahamed Ahanger, Anil Audumbar Pise, Rajnish Ratna

**Affiliations:** ^1^Woxsen University, Hyderabad, Telangana, India; ^2^School of Business Studies, Sharda University, Greater Noida, India; ^3^TAPMI School of Business Studies, Manipal University, Jaipur, India; ^4^Dr Ambedkar Institute of Management Studies, Bangalore, India; ^5^Woxsen University, Hyderabad, Telangana, India; ^6^College of Computer Engineering and Science, Prince Sattam Bin Abdulaziz University, Al-Kharj, Saudi Arabia; ^7^School of Computer Science and Applied Mathematics, University of the Witwatersrand, Johannesburg, South Africa; ^8^Department of Sustainable Engineering, Saveetha School of Engineering, Saveetha Institute of Medical and Technical Sciences, Saveetha University, Saveetha Nagar, Thandalam, Chennai 602105, Tamil Nadu, India; ^9^Gedu College of Business Studies, Royal University of Bhutan, Thimphu, Bhutan

## Abstract

Artificial intelligence is an emerging technology that revolutionizes human lives. Despite the fact that this technology is used in higher education, many professors are unaware of it. In this current scenario, there is a huge need to arise, implement information bridge technology, and enhance communication in the classroom. Through this paper, the authors try to predict the future of higher education with the help of artificial intelligence. This research article throws light on the current education system the problems faced by the subject faculties, students, changing government rules, and regulations in the educational sector. Various arguments and challenges on the implementation of artificial intelligence are prevailing in the educational sector. In this concern, we have built a use case model by using a student assessment data of our students and then built a synthesized using generative adversarial network (GAN). The dataset analyzed, visualized, and fed to different machine learning algorithms such as logistic Regression (LR), linear discriminant analysis (LDA), K-nearest neighbors (KNN), classification and regression trees (CART), naive Bayes (NB), support vector machines (SVM), and finally random forest (RF) algorithm and achieved a maximum accuracy of 58%. This article aims to bridge the gap between human lecturers and the machine. We are also concerned about the psychological emotions of the faculty and the students when artificial intelligence takes control.

## 1. Introduction

In today's world, if anything that is growing other than the stock market is the trend of automating all the business activities. Automation with no or less human interference is the key trend that has been boosting for a decade. As per the expert predictions in 90s, complete automation was expected in late 2010, but as we have seen, it is not that easy for automation to come into the picture. Even seeing the current trend, we can expect total automation in no less than the coming 3 or 4 decades. However, the major business activities and the key and impacting functions are already automated or in the process of automation [1–5]. The biggest and the nearest total automation that we can see the first is the marketing function of the business. The introduction of artificial intelligence is the first key to reduce errors in any process. For any process to reduce errors needs lots of practice, similarly artificial intelligent systems also need lots of data to train themselves for becoming an error-free system or process. For developing artificially intelligent systems, we need to develop algorithms by making use of machine learning languages like Python, R, Julia, etc. Some of the strong literature focuses on the similar problem statement: Jackson Cossitt presents the implementation and validation part of the intelligent tutoring system for a course of a time or a period of study and they could not identify the presence of any longitudinal studies. Huang and Chen [6] discusses various models utilized in the intelligent tutoring process especially on student's knowledge level, learning pedagogy, IQ level, skills, performance analysis, and teaching methodologies, etc.. Dasic et al. [7] made an analytical literature review of intelligent tutoring systems and discussed its execution and usage with concepts of modern learning. Lodhi et al. [8] briefs about the teaching-learning experiences in the sector of education and also they formulated StuA, (student assistant) which fosters the need of newcomers while they join a college by answering their anxieties about faculties, level of evaluations, extra curriculum activities, services offered by libraries and the extracurricular activities prevailing in college, etc. Bond et al. [9] had done a systematic review; in this, they have given a clear idea to widen the research area in artificial intelligence for education. It also helps us to understand the impact of artificial intelligence. Bhattacharya and Nakhare [10] explained progressive research in unraveling the opportunities of AI in the field of vocational courses in the UAE. To be more specific, they focused on how AI tools simulate one-on-one/mentor-mentee and supervised-learning experience to cater to the needs of students in vocational education. Additionally, the study of AI helps to measure the effect of facilitating such an experience on the performance among students. The study makes use of an experimental design to analyze the outcome of an AI-based mentoring system on the achievement of students. It helps to evaluate the success and effectiveness of AI-based mentoring systems to provide better learning outcomes for students. Hasan et al. [11] had recognized effective tutoring system as a capable tutoring system for the upcoming generations by creating channels of emotional recognition, along with computational intelligence.

## 2. Methods

### 2.1. Is AI Needed in Higher Education?

Artificial intelligence is the growing trend, with almost all industries being introduced with artificial intelligence, and even the educational industry is in the process and few of the components of an educational industry being automated, it is time for the exact revolution that we expect in the educational sector [12]. According to human psychology, every person has a different mindset and different capabilities. We have seen that few people are early birds, whereas a few are night owls, a few are outspoken, whereas the others are shy, a few grasp concepts very quick, whereas a few take more time, a few just requires one-time explanation, while the others require huge attention from the tutors, a few have generous questions, while a few have stupid questions. With all these situations being, today's generation think a lot of what others think about them, and they do not try to sum up their problems with a fact that they may sound stupid. Not just the students, but also the tutors face the problems, we have seen that humans tend to have emotions and we do not react always the same, but our one-time reaction could affect some others for a very long-time. We have seen that a few tutors are very rude, and they humiliate students for even silly mistakes. To overcome these problems and to develop strong character in individuals, we need artificial intelligence [13]. Artificial intelligence is the key to developing a better future and protecting individuals from emotional trauma.

### 2.2. AI in Higher Education

#### 2.2.1. Students Perspective

The biggest benefit of introducing artificial intelligence in the education industry is that students would be benefited the most, irrespective of the other benefits that would be received by others in the industry [14–17]. Students would have a tutor that would teach them with the same emotion all the time, he would not be sad, happy, angry, etc. He would be the same all the time. With artificial intelligent tutors, students can learn at their pace, as these systems can be computed according to the student's needs. Students can learn according to their time, as no human has fixed time slots. This would mostly be a single user system, so students would be open to ask doubts, as there are no other students where they feel shy of, or there is no human faculty that they would be scared to face humiliation. Students would be able to question many times, which is not possible for them when human tutors are involved. Students would get clarification of doubts, as and when they arise, irrespective of the traditional education system where doubts are clarified after the class, so the time of other students is not wasted. However, a part of these benefits, there as various flaws that students are going to face, they are going to miss the personal emotions with the tutors. It is said that the student shares all his problems with his tutor and he receives a genuine answer, but with an artificially intelligent system, where there are no emotions, there would not be anybody who is going to help them. All that a student requires for success is motivation, and humans are the best to do that, but with artificially intelligent systems, we cannot expect that [18]. Students are going to miss the fun of childhood, the importance of socializing, the quality of adaptability, the knowledge of understanding other humans, and the importance of emotions because all they are going to do is the study and learn from a teacher that is designed to teach them academic concepts, with everyone studying at their own pace, time, and space.

#### 2.2.2. Teacher's Perspective

**Figure 1:**
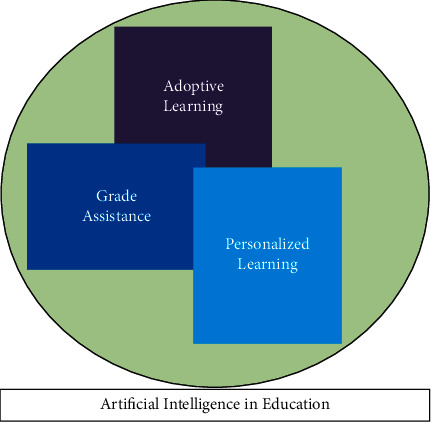
Artificial intelligence's role in the educational sector.

When looking from a perspective of a teacher, they are the one who is at a loss of job. They are the ones who is going to lose their livelihood. When we see that involvement of artificially intelligent systems requires lots of human data, but what is next after the data are collected by the systems [19–21]. The artificial intelligent systems would train themselves with the data of the existing teachers and would then be implemented to do the same task of what is being done by the human tutors. Human tutors that too a few would be left with handling the student's data, and checking the compatibility of the artificially intelligent system. However, as we know that humans are emotionally driven, we would see that the teachers are being promoted back, and students tend to incline towards human tutors, at least till the time we develop emotional artificial intelligent systems. Human tutors would be the need of the upcoming future.  Figure 1 shows clear information about AI's importance in the Education sector, from in student's perspective and as well-teaching staff's perspective [22, 23].

### 2.3. Transformation of Artificial Intelligence in Higher Education

Transformation is not an easy process for any industry, and with the education industry, it is the most difficult as it is very diversified and at the same time, it is very vast. Total transformation of the education industry would be a step-wise process, and there would be various testing models before implementation. Implementation of applicability of intelligent retrieval in the educational sector is also a huge cost-driven concept, with no guaranteed returns of success. Though the transformation is needed we cannot predict the fact whether is it good. Transformation of the education industry is going to be difficult, because of the fact of acceptability by the students, as various educational institutions receive admissions because of the faculties that teach at their institute. The concept of transformation

lies with the urge to satisfy the human needs; probably, there are a few renowned faculties whom every student wants to learn from, but with the time element those few faculties cannot teach everyone, and also most of them will not be able to afford the cost of such faculties. With the transformation being brought, it is the cost that is the highest matter of concern because not everyone will be willing to pay that amount, and profits of those invested would generate negative returns in the short run [8, 24]. The biggest drawback of transformation is accessibility by individuals, as such systems would require huge computational power to function, and the availability of such computational power is limited.

### 2.4. Application of AI in Higher Education-How AI Personalizes the Higher Education System?

**Figure 2:**
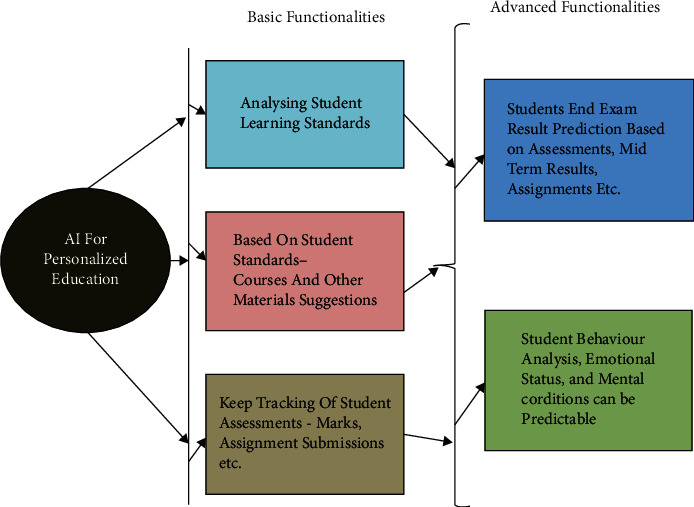
Ai personalizes higher education system.

Artificial intelligent systems would of course help the students a lot. Students can customize the tutors according to their needs of pace, time, and space. Every student from the world has a different language, and at the same time, the influence of the home language is also high. It would be difficult for a person from India to understand the English of a tutor from America; therefore, artificial intelligence can help them to remove this barrier, where they can customize the same concept. At the same time, a few people prefer to understand a thing in their mother tongue, artificial intelligence can customize the lectures, according to the language requirements of individuals. A few people are slow at grasping things, they can reduce the pace of the tutor to understand things properly. A few are working individuals, they can customize the lecture timings according to their availability, there would be a question that we can go through online lectures that are recorded, but the benefit of having artificial intelligent systems is it would be more realistic, and there would be immediate doubt clarification [25]. There is a traditional education system that gives you limited scope to choose subjects that you want to pursue; but with artificially intelligent systems, we have a chance to customize the education pattern where a person from science can learn accounts; though not having prior knowledge, we have artificial intelligence systems that can be customized to teach them. I, as an individual being driven by emotions tend to take long holidays a few times, and on the other hand, I tend to learn continuously for days, but with a traditional education system even with a lack of interest I have to attend classes, and with huge interest, I have to compromise my study with lack of availability of tutors, this is the place where artificially intelligent systems can work on with the students. Students would be able to question open to the systems, and they would get the answers immediately. We have seen that few tutors are biased with various students, so the others lack their interest in the subject, but with no emotions being driven we would see no bias-ness in the education system that would drive students to work hard. Students would be able to access any concept in the world, even if that is outside their domain, because the systems are virtually connected, and has the highest knowledge. The traditional concept of learning and writing exams would also be changed, and probably students would be facing real-life scenarios with the transformation of the education system.  Figure 2 shows how AI is changing the personal education limits from basic functionalities to advanced levels.

### 2.5. Ethical Thinking

The biggest drawback of implementing the artificially intelligent system soon is the lack of emotional intelligence in them. We have seen that human tutors try to develop the right attitude toward looking at things. For example, a human tutor would explain the concept of bomb creation, but he will limit the scope of explanation and also, he will try to develop ethics and intelligence of discriminating between good and bad in the students, he will always try to help them with their negative feelings. We cannot expect the same from artificially intelligent systems because they lack emotions, and they would not even try to go further extent to help individuals, and with the virtual connection, there would be no limit to the information that an individual can access to without knowing his intentions.

At the same time, human brains can easily be controlled; in an article that I read stated that Facebook shut down two of its robots because they were trying to communicate in a language of their own, and suppose that happens with the educational system, it can create havoc as it would be accessed by most of the individuals and it, having the knowledge of controlling human brains, it can spread wide destruction in the world. We do not know how the emotional artificial systems would act, whether they would be positive or negative, whether they can help the students or not, whether the concept of ethics can be created with the help of these systems in individuals or not. Suppose the systems work according to design it would be the biggest grant for the human race, if not vice versa [26, 27]. The concept of development of ethics in these systems is more important than developing them in individuals as the teacher has to learn first, only then can he teach it to others. Ethical thinking is necessary for both the systems and individuals, with a fact that individuals can understand to limit their needs and accessibility, and systems can understand to limit their scope of providing knowledge and developing character in individuals [28, 29]. With all these, the developers of these systems should also be ethical to limit the scope to a certain extent, rather than just concentrating on the reach of customers.

## 3. Results Based on Use-Case: Predictive Analytics on Assessment Module

On this problem statement, we have done a literature survey and found a good dataset from Open University Learning Analytics Dataset (OULAD) [30, 31]. Researchers Kuzilek et al. have done significant work on the dataset in terms of data modeling, data analysis, and data visualization. From that ideology, we have considered assessment modeling and came out with our strategy to build a student assessment modeling system using the synthetic data creation concept. We have used the synthetic data vault (SDV) library to generate synthetic data. We have considered our student's data from Woxsen University, the school of business, specialization of artificial intelligence and machine learning, and also added a few student's data from the computer science department and general MBA students data mentioned as HR. So, that we have generated synthetic data from the original data. Here, we have adopted a mechanism of GAN modeling, i.e., a generative adversarial network (GAN) model to tabulate the student data [32, 33].Generative adversarial network (GAN) is a popular deep neural network. GAN is a unsupervised learning task in machine learning. It consists of two models that automatically discover and learn the data patterns in input. The two models are called generator and discriminator strive with each other to analyze, capture, and copy the variations within a data set. We can create new samples using GAN that may have been drawn from the original data set. The generator is a heart of GAN. It is a model that is used to produce examples, and it is one that you should invest in if you want to help attain exceptionally high performance at the conclusion of the training. The purpose of the generator is to be able to generate synthetic instances from a given input. The discriminator is a sort of classifier whose goal is to distinguish between actual and fraudulent data supplied by the computer. Classifiers are not just for finding picture classes; they may also be used to determine video data of any kind, as well as a variety of other things. We have chosen GAN for generating more sample data from less data. In this research we have generated 9000 samples form 114 samples.  Table 1 shows the model of the tabular data given as the input to the GAN model with 114 rows and 10 columns. The data synthesis concept has been used behind the GAN, and using this, 9000 rows of data are generated from 114 rows of input. The synthesized data were analyzed using machine learning algorithms. In machine learning, the support vector machine (SVM) is mainly used for classifying labelled data. The performance of support vector machine is quite impressive for both linear and non-linear classifications. SVM locates a hyperplane that divides the various groups of data. In two-dimensional space, this hyperplane is nothing more than a line to cut the data points. SVM plots every piece of data in the sample in an two-dimensional axis, where N denotes the number of classes in the sample. Next, choose the most appropriate line for splitting the data points. As a result, you have probably noticed that by default, a support vector machine can only do 0 or 1 classification (i.e., choose between two classes).

Random forest: It is an ensemble machine learning model used to deal with classification and regression problems. It employs more than one decision tree for solving complex classification problems.

K-Nearest Neighbor (KNN): It is a machine learning algorithm and it is mainly used for classifying labeled data. It assumes that the new data point and data given for training the model are equivalent and assigns the new data to the group that is neighboring to the prevailing ones. Based on the K value, the classes are categorized. It does not make any data assumptions because it is a non-parametric model. During training the model, the model trained with the training data and when a new data arrives, it categorize it based on the classes trained by the model.

**Figure 3:**
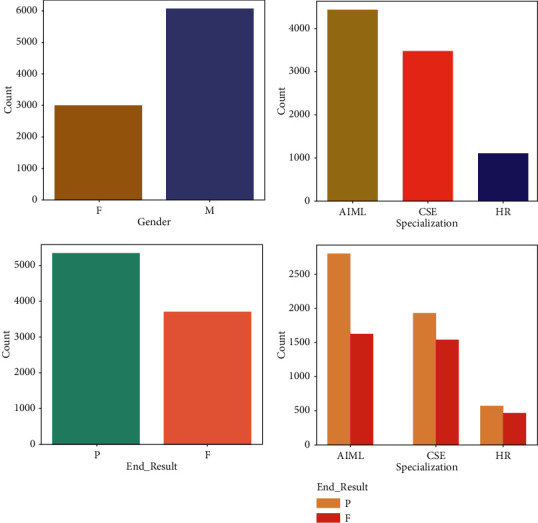
Data modeling on assessment.

In Table 1, a total of 10 attributes are there, namely, Student ID–represents students unique number, Gender-sex details, Department–from the university, student belongs to which department, Specialization–from the Department, Subject code–unique code for each subject from different semesters, Q1, Q2, Q3, and Q4, intermediate and midterm marks, and End Result-final Result as the target variable. After synthesizing the dataset, the data modeling, pattern visualization, and feature extraction and analysis is done [34, 35].  Figure 3 shows the gender, specialization, specialization vs result, and overall result of the entire synthesized dataset.

**Figure 4:**
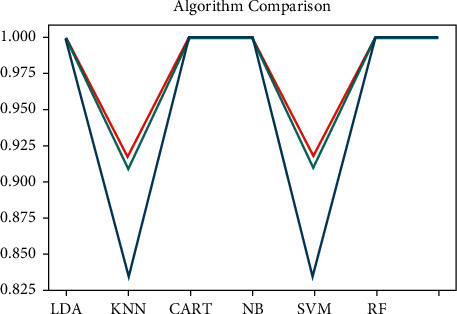
Prediction using algorithms on assessment data, initial condition.

At the initial condition, Figure 4 shows that predictive modeling was applied to the primary input data using machine learning algorithms and achieved 99% of accuracy.

**Figure 5:**
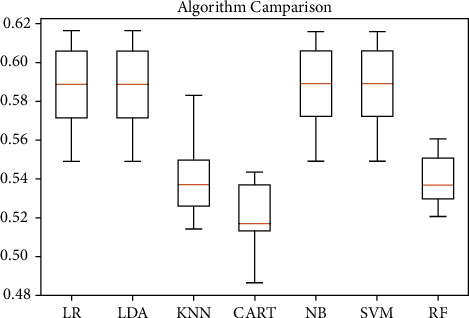
Prediction using algorithms on assessment data, final condition.

**Figure 6:**
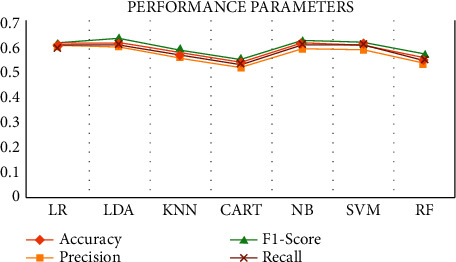
Algorithms performance parameters.

Here, logistic regression (LR), linear discriminant analysis (LDA), K-nearest neighbors (KNN), classification and regression trees (CART), naive Bayes (NB), support vector machines (SVM), and finally, random forest (RF) algorithms [36–40] have been used to predict the end result based on the intermediate test performance values, i.e., midterm marks (Q1, Q2, Q3, and Q4). For the synthesized data, always like 50% of the chances of getting accurate information, the same thing we learned from this research.  Figure 5 shows the final result of synthesized data. Based on the algorithmic analysis, a maximum of 58% of accuracy was achieved. Algorithms performance parameters are shown in Figure 6.

## 4. Conclusion

There is a need for the development of emotional artificial intelligent systems that can be customized by the students according to their own pace, time, and space, but also the systems should be capable enough to help individuals with their emotional trauma. The developer of these systems should be ethical and the developed system should be one, such that it can develop the same in the users. There would be vast knowledge that can help individuals a lot, not limiting their scope to one extent. The systems are cost-driven with no guarantee of returns, but with negative returns for sure in the short run. The acceptability of the individuals is also not sure for such transformations. Here, we have presented a use case for the student assessment system using AI modeling; GAN's and Machine learning algorithms have been used for predictive modeling. With the predictive modeling, approx. 58% of accuracy was achieved using supervised learning algorithms.

## Figures and Tables

**Table 1:** Student information-input data to GAN model.

Student ID	Gender	Department	Specialization	Subject code	Q1	Q2	Q3	Q4	End result

1	M	SB	AIML	A7	9	8	2	4	P
2	F	SB	AIML	A7	2	2	8	9	P
3	M	ST	CSE	CS23	1	0	8	9	P
369	F	SB	AIML	A05	9	9	8	7	P
1200	M	SB	AIML	A8	1	1	2	5	F
1204	F	ST	CSE	CS11	9	9	9	9	P
452	M	SB	HR	S22	10	10	9	10	P
3	M	ST	CSE	CS9	8	2	5	7	P
2	F	SB	AIML	A6	1	4	2	5	F

## Data Availability

The data that support the findings of this study are available on request from the corresponding author.
